# Aftereffect and Reproducibility of Three Excitatory Repetitive TMS Protocols for a Response Inhibition Task

**DOI:** 10.3389/fnins.2019.01155

**Published:** 2019-11-05

**Authors:** Gong-Jun Ji, Jun-Jie Wei, Tingting Liu, Dandan Li, Chunyan Zhu, Fengqiong Yu, Yanghua Tian, Kai Wang, Lei Zhang, Panpan Hu

**Affiliations:** ^1^Department of Medical Psychology, Chaohu Clinical Medical College, Anhui Medical University, Hefei, China; ^2^Department of Neurology, The First Affiliated Hospital of Anhui Medical University, Hefei, China; ^3^Anhui Province Key Laboratory of Cognition and Neuropsychiatric Disorders, Hefei, China; ^4^Laboratory of Cognitive Neuropsychology, Collaborative Innovation Centre of Neuropsychiatric Disorder and Mental Health, Hefei, China

**Keywords:** intermittent theta-burst stimulation, reproducibility, response inhibition, stop signal task, transcranial magnetic stimulation

## Abstract

A number of repetitive transcranial magnetic stimulation (rTMS) protocols have been developed for modulating brain function non-invasively. To identify the most powerful one, these protocols have been compared in the context of the motor system. However, to what extent the conclusions could be generalized to high-level functions is largely unknown. In this study, we compared the modulatory effect of three excitatory rTMS protocols on high-level cognition represented by response inhibition ability. Our first experiment revealed that intermittent theta-burst stimulation (iTBS) could significantly improve reaction time in a stop signal task, while 5-Hz and 25-Hz stimuli were ineffective. This iTBS effect was significantly higher than that for the sham simulation and only occurred in the second session of the stop signal task after iTBS in the first experiment. However, this aftereffect of iTBS was not reproduced in the second experiment, indicating high variability across subjects. Thus, on the one hand, our findings indicate that iTBS on the pre-SMA could improve inhibitory control, but on the other hand, the reliability and reproducibility of this effect needs further investigation.

## Introduction

Transcranial magnetic stimulation (TMS) is a non-invasive neural modulation technique with valuable potential in both neuroscience (Bergmann et al., 2016) and clinical studies (Lefaucheur et al., 2014). In particular, it has been shown that repetitive TMS (rTMS) can temporarily modify brain function for minutes to hours (Iyer et al., 2003; Huang et al., 2005; Hamada et al., 2007; Jung et al., 2008). Stimulations at low (≤1 Hz) and high (≥5 Hz) frequency can increase or decrease neuronal excitability, respectively. Among rTMS protocols, theta-burst stimulation (TBS) can produce long aftereffects (>20 min) using relatively short-term stimulation (typically, 40–190 s) (Huang et al., 2005). A motor-evoked-potential (MEP) study indicated that repeating intermittent TBS (iTBS) three times could further increase the aftereffects (Nettekoven et al., 2014). To identify the protocol with the best modulatory capacity, different stimulation sequences have been compared in the context of motor systems (Zafar et al., 2008; Ji et al., 2017). However, to what extent a conclusion could be generalized to high-level functions is largely unknown. For instance, the dynamic aftereffect of rTMS on cognitive function has rarely been investigated.

Response inhibition represents a key executive function (Aron, 2007; Mirabella, 2014; Hampshire and Sharp, 2015). Specifically, response inhibition refers to the ability to suppress responses that are no longer required or that are inappropriate, such as braking when an animal suddenly crosses the road. In a laboratory setting, a stop signal task (SST) is one of the most effective paradigms for investigating response inhibition of pre-planned movement (Logan et al., 2014). Neuroimaging studies have shown that core regions underlying response inhibition mainly include the bilateral inferior frontal cortex (IFC), pre-supplementary motor area (SMA), globus pallidus, striatum, and subthalamic nucleus (STN) (Aron and Poldrack, 2006; Aron et al., 2007; Zandbelt and Vink, 2010; Mirabella et al., 2012, 2013; Swann et al., 2012). Patients with neuropsychological disorders, such as Parkinson’s disease (PD) (Ye et al., 2014, 2015) and obsessive–compulsive disorder (Chamberlain et al., 2006; de Wit et al., 2012), often exhibit deficits in response inhibition. To non-invasively restore this function, the effectiveness of different rTMS protocols on SST has been investigated in independent studies (Obeso et al., 2013; Watanabe et al., 2015; Yang et al., 2018). For instance, Watanabe et al. (2015) found that excitatory and inhibitory rTMS on the right pre-SMA could improve and impair the response inhibition ability, respectively. In contrast, no positive effect of an excitatory protocol was found following application of 10-Hz stimulation on the right IFC (Yang et al., 2018). In disease conditions (e.g., PD), there are many other excitatory protocols (e.g., 5 and 25 Hz) showing potential in improving response inhibition ability (Chou et al., 2015), but the effect on SST has not been investigated or compared in the same context to date.

Here, we hypothesized that excitatory rTMS protocols could improve response inhibition ability and aimed to identify the most effective from several common protocols. According to a previous rTMS study (Watanabe et al., 2015), the right pre-SMA was defined as the stimulation target. In the current study, we estimated and compared the aftereffects of rTMS protocols [i.e., 5-Hz, 25-Hz, intermittent (i) TBS, and sham stimulation] in a single-blind, crossover design. In particular, we designed two SST sessions before and after each protocol to show the onset time and duration of the rTMS effect.

## Materials and Methods

### Experimental Design

**FIGURE 1:**
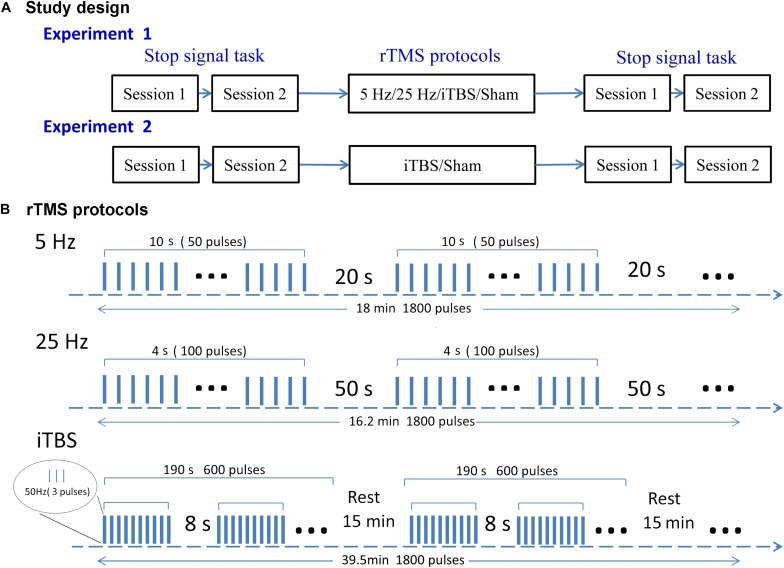
Study design **(A)** and rTMS protocols **(B)**. In experiment 1, each subject received four rTMS protocols on four separate days. Experiment 2 was performed after the end of experiment 1, with subjects receiving iTBS or sham rTMS on two separate days. Both experiments followed a within-subject design. The rTMS protocols were delivered in a randomized order for each subject. Subjects were required to perform the SST twice before and after stimulation. Each rTMS protocol contained 1800 pulses. This takes 18, 16.2, and 39.5 min in the 5-Hz, 25-Hz, and iTBS protocols, respectively.

We used a single-blind, within-subject design to examine the aftereffects of rTMS protocols (Figure 1A). Subjects were blinded to the sequence of the stimulation protocols until the end of the study. There were two experiments. In the first, each subject received four types of rTMS stimulation (5 Hz, 25 Hz, iTBS, and sham) on independent days at least 1 week apart to avoid any carryover effect. The order of the rTMS protocols was randomized within each subject. Before and immediately after each stimulation, participants performed SST for two sessions. The second experiment was designed to validate the findings of the first one in an independent sample. Subjects received two types of rTMS stimulation. One was the most effective protocol in the first experiment, and the other was sham stimulation (the rTMS protocols are shown in Figure 1B). For a study with a random design, it is important to test to what extent participants are able to differentiate sham and real TMS. Here, it took an average of 4 weeks for each subject to complete experiment 1. Owing to the long delay between protocols, the answers given by participants at the end of experiment 1 would not have been accurate. Thus, we did not include a post-experiment questionnaire in the design. For consistency, a post-experiment questionnaire was not included after experiment 2 either.

### Subjects

From May 2017 to April 2018, we recruited 43 healthy, right-handed undergraduates with no history of neurological or psychiatric diseases and no experience in TMS. Of these, 20 and 18 participants completed the first and second experiments, respectively. All participants met the safety criteria for functional fMRI and rTMS, and gave informed consent before participating in the study (Rossi et al., 2009). The study was performed according to the Declaration of Helsinki (2008 revision) and approved by the local ethics committee.

### Stop Signal Task

Response inhibition was assessed with the SST compiled and executed using E-prime 2.0 (Publisher: Psychology Software Tools, Inc., Pittsburgh, PA, United States). One SST session included 200 trials consisting of “Go” (75%) and “Stop” (25%) tasks. During the experiment, participants were instructed to pay attention to a white circle (∼2.5°× 2.5° visual angle) displayed at the center of a 14-inch Dell computer screen. After a black background (1000 ms) and white circle (randomized between 200 and 1000 ms), a white arrow was presented at the center of the circle. In the “Go” trials, the color of the arrow did not change. According to the orientation of the arrow (left or right), participants were instructed to press “F” or “J” on the keyboard using the left or right index finger, respectively. The arrow disappeared upon pressing a button or after 800 ms was elapsed, and the trial terminated. In the “Stop” trials, the color of the arrow changed to red after a so-called stop-signal delay (SSD); participants were asked to withhold the button press. The trials were terminated if the button was mistakenly pressed or at 800 ms after the appearance of the red arrow. The SSD varied among the stop trials according to a staircase procedure (initial SSD, 250 ms): when participants withheld or continued their press response, the SSD was increased or decreased by 50 ms. The participants were instructed to quickly respond to “Go” signals but also to keep in mind that occasional “Stop” signals could appear. To ensure that subjects understood the rule, 100 practice trials were performed before giving the actual test.

### Transcranial Magnetic Stimulation

Transcranial magnetic stimulation was performed using a Magstim Rapid^2^ stimulator (Magstim Company, Whitland, United Kingdom) coupled to a frameless stereotactic optical tracking neuronavigation system (Brainsight; Rogue Research, Montreal, QC, Canada). High-resolution anatomical images were acquired for neuronavigation (repetition/echo time, 8.16/3.18 ms; flip angle, 12; field of view, 256 mm^2^ × 256 mm^2^; 256 × 256 matrix; section thickness, 1 mm, without intersection gap; voxel size, 1 mm^3^ × 1 mm^3^ × 1 mm^3^; 188 sections). The resting motor threshold (RMT) was estimated for each subject to set the individualized stimulation strength before the first rTMS. To measure RMT, MEP amplitudes were recorded for the abductor pollicis brevis muscle using Ag/AgCl surface electrodes when the left “hand knob” area was stimulated with a 70-mm figure-of-eight coil (Magstim Company). The electromyography (EMG) signal was amplified, digitized, and displayed on a computer screen by the Rogue EMG device. RMT was defined as the lowest intensity evoking a small response (>50 μV) in at least 5 of 10 consecutive trials (Ji et al., 2017; Chen et al., 2018).

The repetitive transcranial magnetic stimulation was delivered to the right pre-SMA at Montreal Neurological Institute coordinates (6,6,62) (Watanabe et al., 2015). Each rTMS protocol contained 1800 pulses. The 5-Hz rTMS delivered 5-Hz stimulations at 110% RMT for 18 min (each 10-s stimulation followed by a 20-s rest). The 25-Hz rTMS delivered 25-Hz stimulations at 110% RMT for 16.2 min (each 4-s stimulation followed by a 50-s rest). A typical iTBS protocol lasted for 190 s and consisted of a burst of three pulses delivered at 50 Hz (70% RMT) for 2 s, which was repeated every 8 s for a total of 600 pulses. To achieve a cumulative aftereffect, three typical iTBS were delivered three times at intervals of 15 min (Volz et al., 2013; Nettekoven et al., 2014; Thimm and Funke, 2015; Ji et al., 2017). We used a sham coil that had the same appearance as a real coil and produced a similar sound (Magstim Company). The sham stimulus in the first experiment was the same as the 5-Hz rTMS. Given that iTBS was the most effective protocol, the sham stimulus in the second experiment was the same as for iTBS but delivered via the placebo coil.

### Statistical Analysis

The stop signal reaction time (SSRT) was the primary outcome. According to the standard Race Model (Logan et al., 2014), SSRT was computed by subtracting the average SSD from the mean reaction time (RT) in the “Go” trials. To compare the modulatory capacity of the three rTMS protocols, we first estimated the effect of each active stimulation on SSRT in the first experiment using paired *t*-tests. The first and second sessions after rTMS were compared to the first and second ones before rTMS, respectively (Ji et al., 2017). As a result, we ran six paired *t*-tests and corrected the *P* value according to Bonferroni. Only protocols showing a significant aftereffect were compared to identify the most effective one. The most effective protocol was adopted in the second experiment for the reproducibility test. RT in the “Go” trials was also analyzed as a complementary measure.

## Results

All 23 participants completed the first experiment, but three were excluded because their SSRT exceeded three standard deviations of the mean value in one of the four protocols. Thus, 20 participants were finally included for the first experiment (nine males; mean age ± SD, 21.6 ± 1.0 years). In the second experiment, 20 subjects were initially recruited and 18 (13 males; mean age ± SD, 22.1 ± 2.5) completed the experiment. No serious side effect was reported. Several subjects reported that the noise was too loud and that their scalp felt uncomfortable, but that these effects disappeared after the stimulation was finished.

### Experiment 1

**FIGURE 2:**
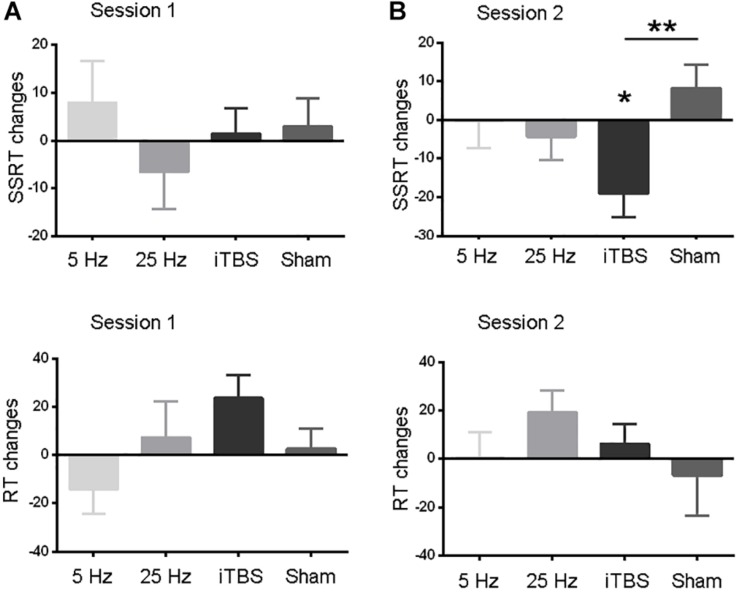
Comparisons of behavior measures within and between protocols in sessions 1 **(A)** and 2 **(B)**. RT and SSRT changes (*Y* axis) were computed by subtracting pre-rTMS from post-rTMS values. Data are shown as the mean and SEM. Asterisks indicate a significant within-protocol aftereffect. ^∗^*P* < 0.05, ^∗∗^*P* < 0.01.

The baseline measures (RT and SSRT) were similar between the two sessions before each protocol (paired *t*-tests, all *P* > 0.05; Table 1). The primary outcome, SSRT, did not show significant changes in the first session (Figure 2A and Supplementary Table S1). In the second session, only iTBS showed significant effects on SSRT (*t* = 3.2, *P* = 0.03; Figure 2B and Supplementary Table S1). In comparison to the sham protocol, the iTBS protocol induced larger SSRT (*t* = 3.1, *P* = 0.006; Figure 2B) changes during the second session. No significant RT alteration was found in the first (Figure 2A) or second (Figure 2B) session after iTBS, 5-Hz, or 25-Hz rTMS (Supplementary Table S1).

**TABLE 1:** Stop signal measures in the first experiment.

	**Session 1**		**Session 2**	
	**RT (ms)**	**SSRT (ms)**	**RT (ms)**	**SSRT (ms)**
**5 Hz**				
Pre	479 ± 22	231 ± 8	467 ± 22	237 ± 6
Post	465 ± 22	239 ± 7	468 ± 24	237 ± 8
Cohen’s *d*	0.31	0.21	0.009	0.008
Power	0.26	0.15	0.05	0.05
**25 Hz**				
Pre	468 ± 18	246 ± 6	468 ± 21	236 ± 5
Post	475 ± 21	239 ± 8	488 ± 23	232 ± 6
Cohen’s *d*	0.11	0.19	0.47	0.17
Power	0.07	0.13	0.51	0.11
**iTBS**				
Pre	467 ± 21	245 ± 6	480 ± 22	**255** ± **4**
Post	491 ± 23	246 ± 4	486 ± 23	**236** ± **6**
Cohen’s *d*	0.55	0.06	0.17	0.72
Power	0.65	0.06	0.11	0.86
**Sham**				
Pre	490 ± 21	231 ± 5	490 ± 24	226 ± 6
Post	493 ± 23	234 ± 4	483 ± 20	235 ± 4
Cohen’s *d*	0.07	0.04	0.10	0.31
Power	0.06	0.05	0.07	0.26

*Data are reported as the mean ± SEM. Numbers in bold indicate measures significantly changed after rTMS.*

#### Supplementary Analysis

We also analyzed the SSRT and RT data using a two-way (time by session) repeated-measures analysis of variance (ANOVA) for each protocol independently. Similar to the results reported above, only SSRT in the second session after iTBS showed significant aftereffect. To test whether the iTBS effect in the second session was different from sham stimulation, we performed a two-way [time (levels: pre- and post-rTMS) by protocol (levels: iTBS and sham)] repeated-measures ANOVA on the data from the second session. Significant interaction effect was found, which suggested different aftereffects between iTBS and sham stimulation. In all, these findings were consistent to that found in the main analyses. See details in the section “Supplementary Results” (Supplementary Tables S1, S2).

### Experiment 2

For the subjects in this experiment, there was a similar male/female ratio (Fisher’s exact test, *P* = 0.11), age (*t* = 0.98, *P* = 0.34), and baseline SSRT (session 1, *t* = 1.57, *P* = 0.13; session 2, *t* = 1.71, *P* = 0.09) to those of the first experiment. Interestingly, the RMT was higher for this experiment (mean = 61, SEM = 4.05) than for the first experiment (mean = 52.6, SEM = 1.52; *t* = 1.43, *P* = 0.0003). Similar to the first experiment, we initially estimated the rTMS effect within each protocol. However, a paired *t*-test indicated that the iTBS and sham protocols did not significantly change SSRT or RT in either the first or the second session (Table 2; all *P* > 0.05).

**TABLE 2:** Stop signal measures in the second experiment.

	**iTBS**		***t*/*P***	**Cohen’s *d*/power**	**Sham**		***t*/*P***	**Cohen’s *d*/power**
	**Pre**	**Post**			**Pre**	**Post**		
**Session 1**								
RT (ms)	438 ± 21	426 ± 24	2.08/0.053	0.49/0.5	436 ± 17	428 ± 17	0.46/0.65	0.10/0.07
SSRT (ms)	230 ± 7	232 ± 8	0.27/0.79	0.06/0.05	255 ± 9	245 ± 8	0.74/0.47	0.29/0.21
**Session 2**								
RT (ms)	443 ± 22	424 ± 21	1.98/0.064	0.46/0.45	442 ± 17	430 ± 16	1.05/0.31	0.25/0.17
SSRT (ms)	240 ± 8	239 ± 10	0.12/0.90	0.03/0.05	248 ± 9	239 ± 8	0.69/0.50	0.28/0.2

*Data, mean ± SEM.*

## Discussion

We investigated the effect of three excitatory rTMS protocols on response inhibition by stimulating the pre-SMA. In the first experiment, iTBS significantly improved the SSRT in the SST, while 5-Hz and 25-Hz protocols did not. This effect was significantly greater than that induced by the sham stimulation and only occurred during the second session of the SST after iTBS. However, the effect of iTBS was not reproduced in the second experiment.

In traditional rTMS protocols (e.g., 5 or 25 Hz), thousands of stimulations are necessary to produce a long-term aftereffect (Touge et al., 2001; Peinemann et al., 2004). The stimulations should be separated into a number of trains by several seconds to avoid side effects such as epilepsy (Rossi et al., 2009). As a result, it usually takes more than 10 or 20 min to complete one rTMS session. In contrast, TBS protocols provide a rapid way to establish long-term aftereffects on the motor system (Huang et al., 2005). To test whether the long-term aftereffects of TBS persisted when high-level cognitive functions are considered, we exploited the SST. Among the three real rTMS protocols, only TBS showed significant effects on SSRT. This result is different from previous studies comparing the effects of TBS and conventional protocols on the motor system. For instance, Zafar et al. (2008) found that both the iTBS and 5-Hz protocols significantly elevated the MEP amplitude. These data suggest that TBS may be a more appropriate protocol for high cognition modulation. In addition, the SSRT did not decrease immediately after stimulation, but in the second session, suggesting a time specificity of iTBS modulation. A delayed aftereffect has been reported for both motor and high-level cognition systems. Peinemann et al. (2004) found that rTMS could alter the MEP 1 min after stimulation, but the effect on short-latency intracortical inhibition or facilitation occurred 15 min later. Neuroimaging studies investigating TBS on oculomotor (Hubl et al., 2008) and cognitive control systems (Gratton et al., 2013) also found a delayed aftereffect at 20 min after stimulation. All of these studies suggest that TBS could be a potential treatment protocol for patients diagnosed with diseases associated with inhibition control deficits, such as PD and obsessive–compulsive disorder.

Despite the potential of rTMS in clinical application, it is not considered as a conventional treatment for most mental disorders. A key reason is the variability of the physiological and behavioral responses to rTMS (Hinder et al., 2014; Lopez-Alonso et al., 2014). Thus, we performed the second experiment to test the reproducibility of our findings in the first experiment. However, SSRT was not significantly modulated in either the first session or the second session after iTBS in this second experiment. This inconsistency between the two experiments may be explained by high variability across subjects or sessions in the rTMS studies. iTBS was initially proposed as an excitatory protocol that can increase the cortical excitability of the primary motor cortex (Huang et al., 2005). Although several studies replicated the aftereffect of iTBS (Zafar et al., 2008; Schilberg et al., 2017), others did not (Hamada et al., 2013; Lopez-Alonso et al., 2014). Hamada et al. (2013) found that the aftereffect of TBS was strongly influenced by the interneuron networks that were recruited during stimulation. In addition, more variables need to be matched, such as time of day, attention, and genetics, to obtain robust findings (Ridding and Ziemann, 2010). Notably, most variability studies focused on the motor system; our work further indicates that it is also important to estimate the reliability and reproducibility of any rTMS effect on high cognitive function.

Response inhibition function is associated with areas in the bilateral cortex, such as the SMA, IFC, and STN. Deep brain stimulation studies have indicated that bilateral (van den Wildenberg et al., 2006; Mirabella et al., 2012, 2013) rather than unilateral (Mancini et al., 2018) stimulation of the STN could restore inhibitory control in PD. Behavior studies among patients (Aron et al., 2003; Swick et al., 2008; Mirabella et al., 2017) and healthy subjects (Li et al., 2008) also suggested the importance of the right as well as the left cortex for inhibitory performance. However, in our study, we only modulated the function of the right SMA. This unilateral stimulation may not be strong enough to achieve a stable aftereffect on inhibition control. The reliability and reproducibility of the effect may be increased by stimulating bilaterally the pre-SMA.

Two limitations of the current study should be mentioned. First, the sample size for both experiments was relatively small. Future studies with hundreds of participants may clarify the effect of iTBS on response inhibition (Lopez-Alonso et al., 2014). Second, the baseline RMT was significantly different between participants in the two experiments. Although the effect of this difference could be largely diminished using individualized TMS thresholds, it would be better to pair this baseline parameter between experiments in the future.

## Conclusion

We compared the ability of three rTMS protocols to alter high cognitive function. Response inhibition tasks were selected because of their importance in shaping motor strategies and for its involvement in many neurological diseases. Although the first experiment indicated that iTBS was effective in improving response inhibition, the effect was not reproduced in the second experiment. Thus, on the one hand, our findings indicate that iTBS on the pre-SMA could improve inhibitory control, but on the other hand, the reliability and reproducibility of this effect need further investigation.

## Data Availability Statement

The datasets generated for this study are available on request to the corresponding author.

## Ethics Statement

The studies involving human participants were reviewed and approved by the Anhui Medical University. The participants provided their written informed consent to participate in this study.

## Author Contributions

All authors listed have made a substantial, direct and intellectual contribution to the work, and approved it for publication. LZ and PH had full access to all data in the study, and took responsibility for the integrity of the data and the accuracy of the data analysis. G-JJ, J-JW, and TL conceived and designed the study, acquired, analyzed, or interpreted the data. TL, DL, CZ, PH, and FY performed the administrative, technical, or material support. CZ, FY, YT, and KW supervised the study.

## Conflict of Interest

The authors declare that the research was conducted in the absence of any commercial or financial relationships that could be construed as a potential conflict of interest.

## References

[B1] Aron A. R. (2007). The neural basis of inhibition in cognitive control. *Neuroscientist* 13 214–228. 10.1177/1073858407299288 17519365

[B2] Aron A. R. Behrens T. E. Smith S. Frank M. J. Poldrack R. A. (2007). Triangulating a cognitive control network using diffusion-weighted magnetic resonance imaging (MRI) and functional MRI. *J. Neurosci.* 27 3743–3752. 10.1523/JNEUROSCI.0519-07.2007 17409238 PMC6672420

[B3] Aron A. R. Fletcher P. C. Bullmore E. T. Sahakian B. J. Robbins T. W. (2003). Stop-signal inhibition disrupted by damage to right inferior frontal gyrus in humans. *Nat. Neurosci.* 6 115–116. 10.1038/nn1003 12536210

[B4] Aron A. R. Poldrack R. A. (2006). Cortical and subcortical contributions to stop signal response inhibition: role of the subthalamic nucleus. *J. Neurosci.* 26 2424–2433. 10.1523/JNEUROSCI.4682-05.2006 16510720 PMC6793670

[B5] Bergmann T. O. Karabanov A. Hartwigsen G. Thielscher A. Siebner H. R. (2016). Combining non-invasive transcranial brain stimulation with neuroimaging and electrophysiology: current approaches and future perspectives. *Neuroimage* 140 4–19. 10.1016/j.neuroimage.2016.02.012 26883069

[B6] Chamberlain S. R. Fineberg N. A. Blackwell A. D. Robbins T. W. Sahakian B. J. (2006). Motor inhibition and cognitive flexibility in obsessive-compulsive disorder and trichotillomania. *Am. J. Psychiatry* 163 1282–1284. 10.1176/appi.ajp.163.7.1282 16816237

[B7] Chen X. Ji G. J. Zhu C. Bai X. Wang L. He K. (2018). Neural correlates of auditory verbal hallucinations in schizophrenia and the therapeutic response to theta-burst transcranial magnetic stimulation. *Schizophr. Bull.* 10.1093/schbul/sby054 [Epub ahead of print]. 29733409 PMC6403092

[B8] Chou Y. H. Hickey P. T. Sundman M. Song A. W. Chen N. K. (2015). Effects of repetitive transcranial magnetic stimulation on motor symptoms in Parkinson disease: a systematic review and meta-analysis. *JAMA Neurol.* 72 432–440. 10.1001/jamaneurol.2014.4380 25686212 PMC4425190

[B9] de Wit S. J. de Vries F. E. van der Werf Y. D. Cath D. C. Heslenfeld D. J. Veltman E. M. (2012). Presupplementary motor area hyperactivity during response inhibition: a candidate endophenotype of obsessive-compulsive disorder. *Am. J. Psychiatry* 169 1100–1108. 10.1176/appi.ajp.2012.12010073 23032388

[B10] Gratton C. Lee T. G. Nomura E. M. D’Esposito M. (2013). The effect of theta-burst TMS on cognitive control networks measured with resting state fMRI. *Front. Syst. Neurosci.* 7:124. 10.3389/fnsys.2013.00124 24416003 PMC3874542

[B11] Hamada M. Hanajima R. Terao Y. Arai N. Furubayashi T. Inomata-Terada S. (2007). Quadro-pulse stimulation is more effective than paired-pulse stimulation for plasticity induction of the human motor cortex. *Clin. Neurophysiol.* 118 2672–2682. 10.1016/j.clinph.2007.09.062 17977788

[B12] Hamada M. Murase N. Hasan A. Balaratnam M. Rothwell J. C. (2013). The role of interneuron networks in driving human motor cortical plasticity. *Cereb. Cortex* 23 1593–1605. 10.1093/cercor/bhs147 22661405

[B13] Hampshire A. Sharp D. J. (2015). Contrasting network and modular perspectives on inhibitory control. *Trends Cogn. Sci.* 19 445–452. 10.1016/j.tics.2015.06.006 26160027

[B14] Hinder M. R. Goss E. L. Fujiyama H. Canty A. J. Garry M. I. Rodger J. (2014). Inter- and Intra-individual variability following intermittent theta burst stimulation: implications for rehabilitation and recovery. *Brain Stimul.* 7 365–371. 10.1016/j.brs.2014.01.004 24507574

[B15] Huang Y. Z. Edwards M. J. Rounis E. Bhatia K. P. Rothwell J. C. (2005). Theta burst stimulation of the human motor cortex. *Neuron* 45 201–6. 10.1016/j.neuron.2004.12.033 15664172

[B16] Hubl D. Nyffeler T. Wurtz P. Chaves S. Pflugshaupt T. Luthi M. (2008). Time course of blood oxygenation level-dependent signal response after theta burst transcranial magnetic stimulation of the frontal eye field. *Neuroscience* 151 921–928. 10.1016/j.neuroscience.2007.10.049 18160225

[B17] Iyer M. B. Schleper N. Wassermann E. M. (2003). Priming stimulation enhances the depressant effect of low-frequency repetitive transcranial magnetic stimulation. *J. Neurosci.* 23 10867–10872. 10.1523/jneurosci.23-34-10867.2003 14645480 PMC6740990

[B18] Ji G. J. Yu F. Liao W. Wang K. (2017). Dynamic aftereffects in supplementary motor network following inhibitory transcranial magnetic stimulation protocols. *Neuroimage* 149 285–294. 10.1016/j.neuroimage.2017.01.035 28130194

[B19] Jung S. H. Shin J. E. Jeong Y. S. Shin H. I. (2008). Changes in motor cortical excitability induced by high-frequency repetitive transcranial magnetic stimulation of different stimulation durations. *Clin. Neurophysiol.* 119 71–79. 10.1016/j.clinph.2007.09.124 18039593

[B20] Lefaucheur J. P. Andre-Obadia N. Antal A. Ayache S. S. Baeken C. Benninger D. H. (2014). Evidence-based guidelines on the therapeutic use of repetitive transcranial magnetic stimulation (rTMS). *Clin. Neurophysiol.* 125 2150–2206. 10.1016/j.clinph.2014.05.021 25034472

[B21] Li C. S. Yan P. Sinha R. Lee T. W. (2008). Subcortical processes of motor response inhibition during a stop signal task. *Neuroimage* 41 1352–1363. 10.1016/j.neuroimage.2008.04.023 18485743 PMC2474693

[B22] Logan G. D. Van Zandt T. Verbruggen F. Wagenmakers E. J. (2014). On the ability to inhibit thought and action: general and special theories of an act of control. *Psychol. Rev.* 121 66–95. 10.1037/a0035230 24490789

[B23] Lopez-Alonso V. Cheeran B. Rio-Rodriguez D. Fernandez-Del-Olmo M. (2014). Inter-individual variability in response to non-invasive brain stimulation paradigms. *Brain Stimul.* 7 372–380. 10.1016/j.brs.2014.02.004 24630849

[B24] Mancini C. Modugno N. Santilli M. Pavone L. Grillea G. Morace R. (2018). Unilateral stimulation of subthalamic nucleus does not affect inhibitory control. *Front. Neurol.* 9:1149 10.3389/fneur.2018.01149 PMC6330317 30666229

[B25] Mirabella G. (2014). Should i stay or should i go? conceptual underpinnings of goal-directed actions. *Front. Syst. Neurosci.* 8:206. 10.3389/fnsys.2014.00206 25404898 PMC4217496

[B26] Mirabella G. Fragola M. Giannini G. Modugno N. Lakens D. (2017). Inhibitory control is not lateralized in Parkinson’s patients. *Neuropsychologia* 102 177–189. 10.1016/j.neuropsychologia.2017.06.025 28647437

[B27] Mirabella G. Iaconelli S. Modugno N. Giannini G. Lena F. Cantore G. (2013). Stimulation of subthalamic nuclei restores a near normal planning strategy in Parkinson’s patients. *PLoS One* 8:e62793. 10.1371/journal.pone.0062793 23658775 PMC3643906

[B28] Mirabella G. Iaconelli S. Romanelli P. Modugno N. Lena F. Manfredi M. (2012). Deep brain stimulation of subthalamic nuclei affects arm response inhibition in Parkinson’s patients. *Cereb. Cortex* 22 1124–1132. 10.1093/cercor/bhr187 21810782

[B29] Nettekoven C. Volz L. J. Kutscha M. Pool E. M. Rehme A. K. Eickhoff S. B. (2014). Dose-dependent effects of theta burst rTMS on cortical excitability and resting-state connectivity of the human motor system. *J. Neurosci.* 34 6849–6859. 10.1523/JNEUROSCI.4993-13.2014 24828639 PMC4019799

[B30] Obeso I. Cho S. S. Antonelli E. Houle S. Jahanshahi M. Ko J. H. (2013). Stimulation of the Pre-sma influences cerebral blood flow in frontal areas involved with inhibitory control of action. *Brain Stimul.* 6 769–776. 10.1016/j.brs.2013.02.002 23545472

[B31] Peinemann A. Reimer B. Loer C. Quartarone A. Munchau A. Conrad B. (2004). Long-lasting increase in corticospinal excitability after 1800 pulses of subthreshold 5 Hz repetitive TMS to the primary motor cortex. *Clin. Neurophysiol.* 115 1519–1526. 10.1016/j.clinph.2004.02.005 15203053

[B32] Ridding M. C. Ziemann U. (2010). Determinants of the induction of cortical plasticity by non-invasive brain stimulation in healthy subjects. *J. Physiol.* 588 (Pt 13), 2291–2304. 10.1113/jphysiol.2010.190314 20478978 PMC2915507

[B33] Rossi S. Hallett M. Rossini P. M. Pascual-Leone A. (2009). Safety, ethical considerations, and application guidelines for the use of transcranial magnetic stimulation in clinical practice and research. *Clin. Neurophysiol.* 120 2008–2039. 10.1016/j.clinph.2009.08.016 19833552 PMC3260536

[B34] Schilberg L. Schuhmann T. Sack A. T. (2017). Interindividual variability and intraindividual reliability of intermittent theta burst stimulation-induced neuroplasticity mechanisms in the healthy brain. *J. Cogn. Neurosci.* 29 1022–1032. 10.1162/jocn-a-01100 28129054

[B35] Swann N. C. Cai W. Conner C. R. Pieters T. A. Claffey M. P. George J. S. (2012). Roles for the pre-supplementary motor area and the right inferior frontal gyrus in stopping action: electrophysiological responses and functional and structural connectivity. *Neuroimage* 59 2860–2870. 10.1016/j.neuroimage.2011.09.049 21979383 PMC3322194

[B36] Swick D. Ashley V. Turken A. U. (2008). Left inferior frontal gyrus is critical for response inhibition. *BMC Neurosci.* 9:102. 10.1186/1471-2202-9-102 18939997 PMC2588614

[B37] Thimm A. Funke K. (2015). Multiple blocks of intermittent and continuous theta-burst stimulation applied via transcranial magnetic stimulation differently affect sensory responses in rat barrel cortex. *J. Physiol.* 593 967–985. 10.1113/jphysiol.2014.282467 25504571 PMC4398532

[B38] Touge T. Gerschlager W. Brown P. Rothwell J. C. (2001). Are the after-effects of low-frequency rTMS on motor cortex excitability due to changes in the efficacy of cortical synapses? *Clin. Neurophysiol.* 112 2138–2145. 10.1016/s1388-2457(01)00651-4 11682353

[B39] van den Wildenberg W. P. van Boxtel G. J. van der Molen M. W. Bosch D. A. Speelman J. D. Brunia C. H. (2006). Stimulation of the subthalamic region facilitates the selection and inhibition of motor responses in Parkinson’s disease. *J. Cogn. Neurosci.* 18 626–636. 10.1162/jocn.2006.18.4.626 16768365

[B40] Volz L. J. Benali A. Mix A. Neubacher U. Funke K. (2013). Dose-dependence of changes in cortical protein expression induced with repeated transcranial magnetic theta-burst stimulation in the rat. *Brain Stimul.* 6 598–606. 10.1016/j.brs.2013.01.008 23433874

[B41] Watanabe T. Hanajima R. Shirota Y. Tsutsumi R. Shimizu T. Hayashi T. (2015). Effects of rTMS of pre-supplementary motor area on fronto basal ganglia network activity during stop-signal task. *J. Neurosci.* 35 4813–4823. 10.1523/JNEUROSCI.3761-14.2015 25810512 PMC6705371

[B42] Yang C. C. Khalifa N. Vollm B. (2018). Excitatory repetitive transcranial magnetic stimulation applied to the right inferior frontal gyrus has no effect on motor or cognitive impulsivity in healthy adults. *Behav. Brain Res.* 347 1–7. 10.1016/j.bbr.2018.02.047 29505803

[B43] Ye Z. Altena E. Nombela C. Housden C. R. Maxwell H. Rittman T. (2014). Selective serotonin reuptake inhibition modulates response inhibition in Parkinson’s disease. *Brain* 137 (Pt 4), 1145–1155. 10.1093/brain/awu032 24578545 PMC3959561

[B44] Ye Z. Altena E. Nombela C. Housden C. R. Maxwell H. Rittman T. (2015). Improving response inhibition in Parkinson’s disease with atomoxetine. *Biol. Psychiatry* 77 740–748. 10.1016/j.biopsych.2014.01.024 24655598 PMC4384955

[B45] Zafar N. Paulus W. Sommer M. (2008). Comparative assessment of best conventional with best theta burst repetitive transcranial magnetic stimulation protocols on human motor cortex excitability. *Clin. Neurophysiol.* 119 1393–1399. 10.1016/j.clinph.2008.02.006 18400556

[B46] Zandbelt B. B. Vink M. (2010). On the role of the striatum in response inhibition. *PLoS One* 5:e13848. 10.1371/journal.pone.0013848 21079814 PMC2973972

