# Supplementary material for: Aftereffect and Reproducibility of Three Excitatory Repetitive TMS Protocols for a Response Inhibition Task
Source: Front Neurosci. 2019 Nov 5;13:1155. doi: 10.3389/fnins.2019.01155 (PMC6848026; doi:10.3389/fnins.2019.01155)
Supplement: Supplementary file 1 [file Table_1.docx]

Supplementary Results

We additionally performed two-way (time by session) repeated measures analysis of variance (ANOVA) on SSRT and RT for each protocol (5Hz, 25Hz, and iTBS). Multiple comparisons in post-hoc and simple effect analysis were corrected by Sidak’s correction.

We found significant interaction effect for SSRT in the iTBS (F_1,38_=6.7, P=0.01), but not with the other protocols (5Hz, F_1,38_=0.54, P=0.49; 25Hz, F_1,38_=0.05, P=0.83). Simple effect analysis for iTBS data showed significant effect in the second session (t=3.40, P=0.003), while the effect in the first session was not significant (t=0.27, P=0.96). These results were consistent with that reported in the main text. Time and session effect was not significant in 5Hz and 25Hz groups. Time but not session effect was significant in the iTBS group. See the full statistic and P values in Table S2.

To test whether the iTBS effect in the second session was different from sham stimulation, we performed a two-way (time by protocol) repeated measures ANOVA on the data from the second session. The interaction (F_1,38_=6.67, P=0.01) effect was significant. Simple effect analysis indicated a marginal significant in iTBS (post- vs. pre-iTBS) after Sidak’s correction (*t* = 2.30, P = 0.05), while the change in sham condition was not significant (*t* = 1.35, P = 0.33). The protocol and time effect were found marginal- (F_1,38_=4.14, P=0.05) and non-significant (F_1,38_=0.45, P=0.51), respectively.

No significant interaction effect was found for RT in either 5Hz (F_1,38_=0.99, P=0.32), 25Hz (F_1,38_=0.46, P=0.50) or iTBS (F_1,38_=1.90, P=0.18) group. Positive result was only found for the time effect in iTBS group (F_1,38_=5.58, P=0.02). Post-hoc analysis showed significant RT increase in the first session (t=2.65, P=0.02), but not the second session (t=0.70, P=0.74). This effect in the first session was not significantly different from that of the sham protocol (t = 1.51, P = 0.15). See the full statistic and P values in Table S2.

Table S1 Aftereffects of three rTMS protocols on measures of experiment 1

| **Post- vs pre-rTMS** | **5-Hz** | | **25-Hz** | | **iTBS** | |
| --- | --- | --- | --- | --- | --- | --- |
|  | T | P | T | P | T | P |
| **SSRT** |  |  |  |  |  |  |
| Session 1 | 0.92 | 0.37 | 0.84 | 0.41 | 0.28 | 0.78 |
| Session 2 | 0.04 | 0.97 | 0.74 | 0.47 | 3.22 | 0.005^a^ |
| **RT** |  |  |  |  |  |  |
| Session 1 | 1.39 | 0.18 | 0.49 | 0.63 | 2.50 | 0.03^b^ |
| Session 2 | 0.04 | 0.97 | 2.12 | 0.05 | 0.76 | 0.46 |

All the statistics are uncorrected values.

^a^This value can survive the Bonferroni correction.

^b^This value can not survive the Bonferroni correction.

Table S2 Statistic and P values of two-way ANOVA for each protocol

| Two-way ANOVA | 5-Hz | |  | 25-Hz | |  | iTBS | |
| --- | --- | --- | --- | --- | --- | --- | --- | --- |
|  | F | P |  | F | P |  | F | P |
| **SSRT** |  |  |  |  |  |  |  |  |
| Interaction | 0.54 | 0.49 |  | 0.05 | 0.83 |  | 6.71 | 0.01 |
| Time | 0.47 | 0.50 |  | 1.25 | 0.27 |  | 4.90 | 0.03 |
| Session | 0.04 | 0.84 |  | 1.38 | 0.25 |  | <0.001 | 0.99 |
| **RT** |  |  |  |  |  |  |  |  |
| Interaction | 0.99 | 0.32 |  | 0.46 | 0.50 |  | 1.90 | 0.18 |
| Time | 0.89 | 0.35 |  | 2.28 | 0.14 |  | 5.58 | 0.02 |
| Session | 0.02 | 0.89 |  | 0.05 | 0.82 |  | 0.02 | 0.90 |
